# Electrospun PEO/rGO Scaffolds: The Influence of the Concentration of rGO on Overall Properties and Cytotoxicity

**DOI:** 10.3390/ijms23020988

**Published:** 2022-01-17

**Authors:** Aleksandra Ivanoska-Dacikj, Petre Makreski, Nikola Geskovski, Joanna Karbowniczek, Urszula Stachewicz, Nenad Novkovski, Jelena Tanasić, Ivan Ristić, Gordana Bogoeva-Gaceva

**Affiliations:** 1 Research Centre for Environment and Materials, Macedonian Academy of Sciences and Arts, Krste Misirkov 2, 1000 Skopje, North Macedonia; nenad@pmf.ukim.mk (N.N.); gordana@tmf.ukim.edu.mk (G.B.-G.); 2 Institute of Chemistry, Faculty of Natural Sciences and Mathematics, Ss. Cyril and Methodius University in Skopje, Arhimedova 5, 1000 Skopje, North Macedonia; petremak@pmf.ukim.mk; 3 Institute of Pharmaceutical Technology, Faculty of Pharmacy, Ss. Cyril and Methodius University in Skopje, Majka Tereza 47, 1000 Skopje, North Macedonia; nikola.geshkovski@ff.ukim.mk; 4 Faculty of Metals Engineering and Industrial Computer Science, AGH University of Science and Technology, al. A. Mickiewicza 30, 30-059 Cracow, Poland; jkarbow@agh.edu.pl (J.K.); ustachew@agh.edu.pl (U.S.); 5 Institute of Physics, Faculty of Natural Science and Mathematics, Ss. Cyril and Methodius University in Skopje, Arhimedova 3, 1000 Skopje, North Macedonia; 6 Faculty of Technology, University of Novi Sad, Bulevar cara Lazara 1, 21000 Novi Sad, Serbia; jelenatanasic@uns.ac.rs (J.T.); ivan.ristic@uns.ac.rs (I.R.); 7 Faculty of Technology and Metallurgy, Ss. Cyril and Methodius University in Skopje, Rugjer Bošković 16, 1000 Skopje, North Macedonia

**Keywords:** nanofibrous scaffolds, reduced graphene oxide, polyethylene oxide, electrospinning, cytotoxicity

## Abstract

Reduced graphene oxide (rGO) is one of the graphene derivatives that can be employed to engineer bioactive and/or electroactive scaffolds. However, the influence of its low and especially high concentrations on scaffolds’ overall properties and cytotoxicity has yet to be explored. In this study, polyethylene oxide (PEO)-based scaffolds containing from 0.1 to 20 wt% rGO were obtained by electrospinning. Morphological, thermal and electrical properties of the scaffolds were characterized by SEM, Raman spectroscopy, XRD, DSC and electrical measurements. The diameter of the fibers decreased from 0.52 to 0.19 µm as the concentration of rGO increased from 0.1 wt% to 20 wt%. The presence of rGO above the percolation threshold (5.7 wt%) resulted in a significantly reduced electrical resistivity of the scaffolds. XRD and Raman analysis revealed delamination of the graphene layers (interlayer spacing increased from 0.36 nm to 0.40–0.41 nm), and exfoliation of rGO was detected for the samples with an rGO concentration lower than 1 wt%. In addition, an evident trend of increasing cell viability as a function of the rGO concentration was evidenced. The obtained results can serve as further guidance for the judicious selection of the rGO content incorporated into the PEO matrix for constructing electroactive scaffolds.

## 1. Introduction

Tissue engineering aims to repair, replace and regenerate damaged tissues. It vastly relies on the use of biodegradable scaffolds, which play a key but temporary role. They mimic the extracellular matrix (ECM) by encouraging cell adhesion and interactions, and facilitating nutrient and waste diffusion, but they simultaneously degrade and are gradually replaced by new tissue [1]. Fibrous-structured scaffolds are of particular interest in tissue engineering research due to their similarity to the naturally occurring ECM. One of the most promising techniques for fibrous structure fabrication is electrospinning [2,3,4,5,6,7,8,9]. It is a versatile electrohydrodynamic process, which allows fine adjustment of multiple processing parameters to form predictable ultrathin fibrous structures with the required morphological and topographical features [10,11,12]. Its setup is very simple (consisting of a high-voltage power supply, a syringe pump and a grounded collector) and can be easily implemented not only on a lab scale, but also in large-scale production. Both synthetic (such as polyglycolides [13], polylactides [14], polycaprolactone [13,15] and polyurethane [16,17,18,19]) and natural polymers (such as collagens [20], gelatine [14], alginates [21], chitosans [20,22] and silk fibroin [14]) have been electrospun to be used for biomedical application. The success of electrospun scaffolds in tissue engineering application depends on how closely they mimic the native ECM in terms of composition, structure and porosity [3], but also conductivity [23]. The electrical conductivity of scaffolds could be beneficial especially in enhancing the propagation of both external signals and cell–cell signaling [24]. This stems from the fact that electrical stimulation is a biologically relevant stimulus, particularly for cardiac, neural and skeletal muscle tissue [25]. In order to create scaffolds with enhanced conductivity, composite materials are often created mainly by embedding carbon-based nanofillers [6,23,26] or gold particles [27] in polymeric matrices.

Due to their extraordinary properties, carbon-based nanofillers are extensively studied in applications going from photonics and optoelectronics to biotechnology and nanomedicine. For instance, graphene (a single, thick layer of *sp^2^*-hybridized carbon atoms arranged in a honeycomb lattice) exhibits excellent electrochemical properties, such as high thermal conductivity (above 3 kW/(m·K)), one million times higher capacity than copper and a low redox potential [28]. Moreover, it has a high specific surface area (2630 m^2^·g^−1^) [29], and a high mechanical strength (about 1100 GPa) [30]. Other derivative forms of graphene, which are easier to be mass produced [31], have been investigated in the last decade, mainly graphene oxide (GO) and reduced graphene oxide (rGO). Due to the disrupted *sp^2^* bonding networks, GO is an electrically insulating material [32]. The process of reduction eliminates most of the oxygen-containing functional groups from GO and partially restores the *sp^2^* trigonal hybridization [33], thus leading to enhanced rGO conductivity by several orders of magnitude [33], and improved stability, dispersibility and reactivity [34,35,36].

The growing interest in the biomedical use of graphene and its derivatives has raised the question of their toxicity and triggered more research to improve their biocompatibility, solubility and stability in aqueous media in order to benefit from their superior properties. Research on the cytotoxicity of graphene-based materials has revealed that it depends on their shape, layer thickness, size, concentration, dose, surface properties and surface chemistry and takes place through different mechanisms [37,38,39,40]. Various approaches to eliminate the undesired cytotoxic effect have been employed. The administration of hydrophilic coatings such as poly(ethylene glycol) (PEG) [41,42], poly(vinyl alcohol) (PVA) [43], polyethyleneimine (PEI) [44], hyaluronic acid (HA) [45] and chitosan [46] on the nano-carbon surface, either covalently or noncovalently, has been shown to be a promising strategy. Another simple, one-step method to improve the biocompatibility of graphene-based materials is embedding them in polymer fibers by the electrospinning technique, where the concentration of the nanofillers could be tailored by solution preparation and process parameters.

Among all the different polymeric structures, PEG or PEO (the names are chemically synonymous, referring to an oligomer or polymer of ethylene oxide; PEG has tended to be used to refer to polymers with a molecular mass below 20,000 g·mol^−1^, whereas PEO has been used for longer-chain polymers) has gained excessive attention since it is a US Food and Drug Administration (FDA)-approved, non-toxic, non-immunogenic and non-antigenic polymer with high water solubility [47]. Additionally, the current research results support that administration of PEG on different graphene derivatives’ surfaces reduces their cytotoxicity and improves physiological stability and pharmacokinetic properties [41,42,48]. Moreover, PEO is particularly suitable for electrospinning; it easily dissolves in both organic solvents and aqueous solutions and is often used as a processing aid, allowing electrospinning of materials for which this procedure is normally precluded [49,50], and improving fiber properties and functionalities [51,52].

In this study, electrospun PEO-based scaffolds with different concentrations of rGO (from 0.1 to 20 wt%) were obtained. PEO was selected in order to exploit its advantages: being an easily spinnable material and a polymer that suppresses the cytotoxicity of carbon-based nanofillers. Morphological, thermal and electrical properties of the PEO/rGO scaffolds were characterized by various analytical techniques including Raman spectroscopy, X-ray diffraction (XRD), scanning electron microscopy (SEM), differential scanning calorimetry (DSC) and electrical testing; an in vitro cytotoxicity test was also performed. The results that emerged from this study are envisioned to serve as guidance for the judicious selection of the rGO concentrations incorporated when constructing electroactive scaffolds.

## 2. Results and Discussion

**Figure 1:**
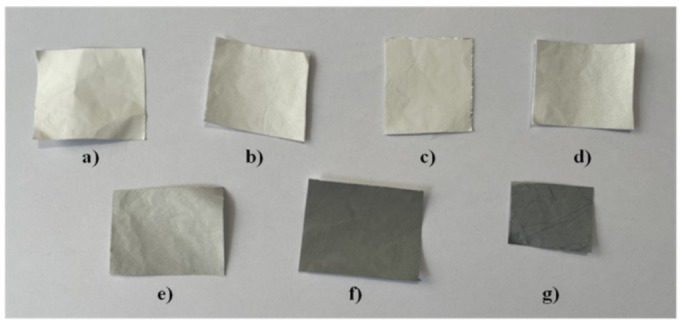
The electrospun PEO scaffolds with different concentrations of rGO: (**a**) PEO-neat, (**b**) PEO-rGO-0.1, (**c**) PEO-rGO-0.5, (**d**) PEO-rGO-1.0, (**e**) PEO-rGO-5.0, (**f**) PEO-rGO-10.0 and (**g**) PEO-rGO-20.0.

Seven scaffolds based on PEO with different concentrations of rGO: 0, 0.1, 0.5, 1.0, 5.0, 10.0 and 20.0 wt%, were successfully fabricated by electrospinning. Figure 1 shows a photograph of the obtained scaffolds indicating the color changes with the increased rGO concentration.

**Figure 2:**
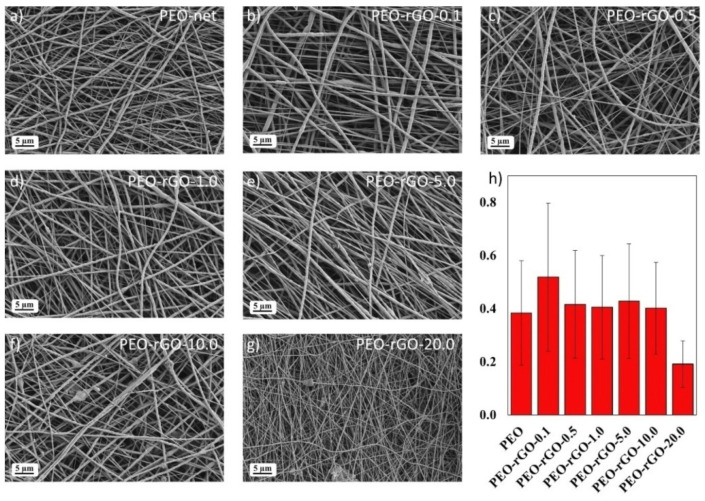
SEM micrographs of electrospun fibers: (**a**) PEO-net, (**b**) PEO-rGO-0.1, (**c**) PEO-rGO-0.5, (**d**) PEO-rGO-1.0, (**e**) PEO-rGO-5.0, (**f**) PEO-rGO-10.0 and (**g**) PEO-rGO-20.0. (**h**) Graph of the average fiber diameters with standard deviations.

The SEM micrographs, showing the morphology of the scaffolds, are presented in Figure 2. It is evident that the microstructure is fibrous and porous, constituted of randomly oriented fibers that form a 3D interconnected porous network, which is very important for tissue engineering application and represents a feasible imitation of the structure of the extracellular matrix (ECM). PEO-neat fibers had an average diameter of 0.38 ± 0.20 µm, and two fractions of fibers could be observed: one with smaller diameters around 300 nm, and another with larger diameters above 600 nm—this is why the standard deviation (SD) is relatively high. The addition of rGO caused an increase in the average fiber diameters, which was apparent for the sample containing the lowest amount of rGO (0.1 wt%), having an average fiber diameter of 0.52 ± 0.28 µm. For the samples containing more than 0.5 wt% rGO, the variations in the fibers’ diameter were insignificant (0.42 ± 0.20 µm for PEO-rGO-0.5; 0.41 ± 0.19 µm for PEO-rGO-1.0; 0.43 ± 0.22 µm for PEO-rGO-5.0; and 0.40 ± 0.17 µm for PEO-rGO-10.0). However, much thinner and more homogeneous fibers with an average diameter of 0.19 ± 0.09µm were obtained by electrospinning of the polymer solution with 20 wt% rGO. This effect of a 50% reduction in the fibers’ diameter compared to the neat PEO is probably due to the improved conductivity of the PEO-rGO solution.

**Figure 3:**
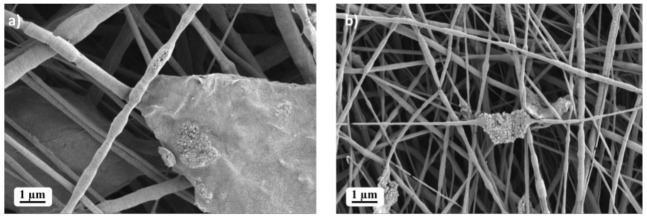
SEM micrographs of (**a**) PEO-rGO-10.0 and (**b**) PEO-rGO-20.0.

The detailed SEM micrographs registered at higher magnification revealed the formation of filler agglomerates for the samples that contain 10 and 20 wt% rGO (Figure 3).

**Figure 4:**
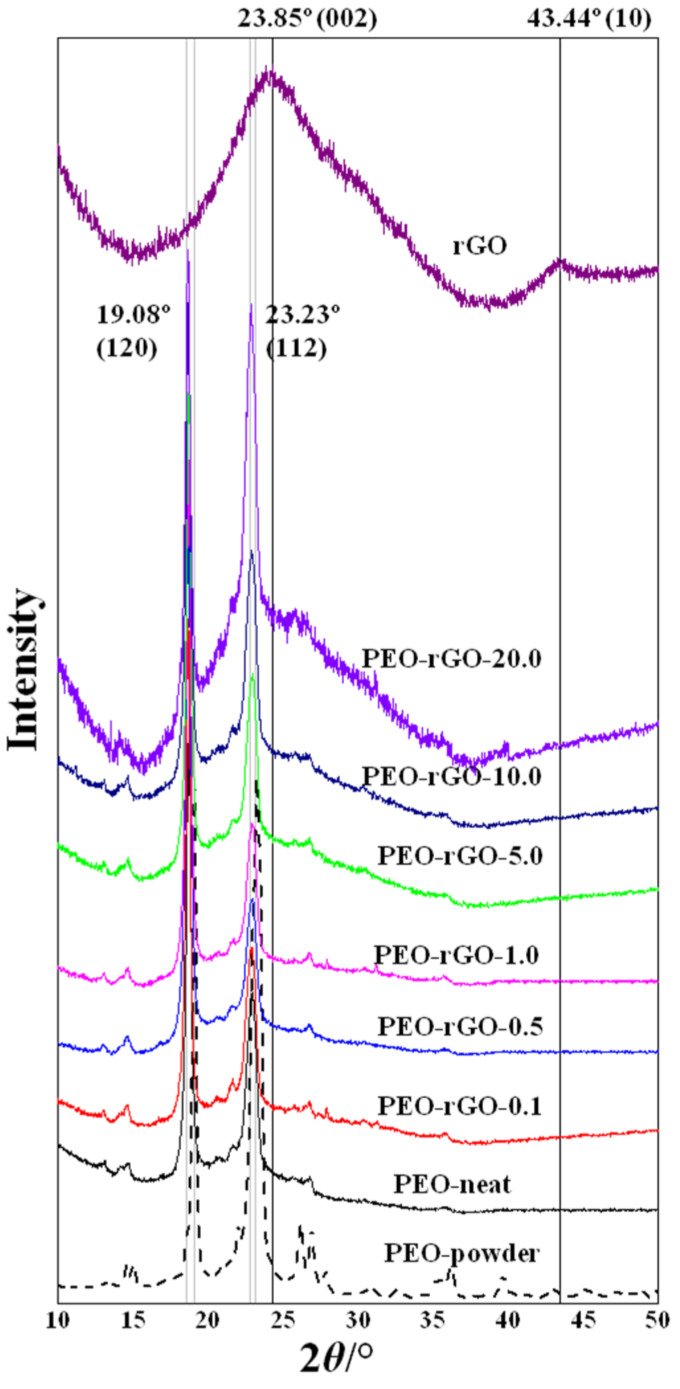
XRD patterns of rGO, PEO-powder, electrospun PEO-neat and electrospun PEO-based samples modified with rGO.

To evaluate the level of dispersion of rGO layers in the PEO matrix, XRD analysis was used. This method is often applied to identify the degree of dispersion of layered nanofillers in polymer matrices. The presence of polymer macromolecules in the interlayer spacing simultaneously increases the degree of dispersion, which is observed as a shift in the diffraction peaks towards lower values of 2*θ*, and decreases the degree of ordering, which is manifested as the appearance of broader peaks with a lower intensity [53,54]. Figure 4 shows the XRD patterns of: rGO-powder, electrospun PEO-neat and PEO/rGO samples with different rGO contents (0.1; 0.5; 1.0; 5.0; 10.0; and 20.0 wt%).

PEO has a monoclinic crystalline structure with its (120) planes parallel to the PEO chain direction and (112) planes intersecting the chain direction [55]; thus, two strong diffraction peaks at 2*θ* = 19.08° and 23.23° emerged from these plains, respectively (Figure 4). Interestingly, the XRD pattern of the PEO-powder sample revealed better-resolved and sharper peaks that exhibited a higher intensity in comparison to the PEO-neat electrospun analogue. In addition, several higher-order reflections emerged that were not evidenced in the XRD pattern of the neat electrospun PEO. This could be explained by the fact that the powdered crystallites are randomly oriented, and the Bragg condition is satisfied for more scattering angles, resulting in a greater number of registered diffraction peaks. In other words, this outcome indicates that the formation of the PEO crystalline microstructure is, to some extent, hindered during electrospinning.

**Figure 5:**
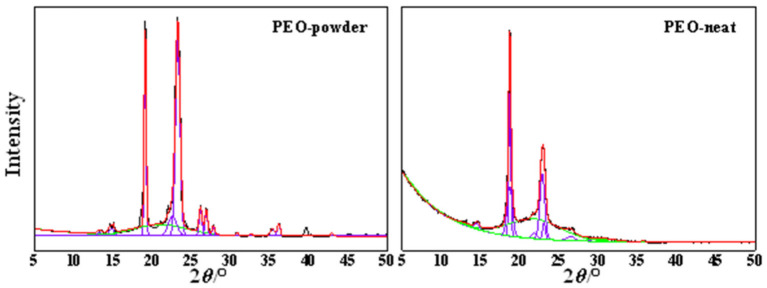
Deconvolution of the XRD patterns of: PEO-powder and electrospun PEO-neat. Violet: crystalline peaks; green: amorphous peaks; red: calculated curve; black: observed curve.

The degree of crystallinity, *χ*_c_, of the PEO-powder and electrospun PEO-neat was determined using Equation (4), as a ratio of the sum of the area of the crystalline XRD peaks (*A*_c_) and the total area of both amorphous (*A*_a_) and crystalline (*A*_c_) XRD peaks. A deconvolution, using the Gaussian function, was undertaken for the peaks relevant for the input of the crystalline and amorphous contributions (Figure 5). The obtained result inferred a 29% reduction in the degree of crystallinity for the electrospun PEO-neat sample in comparison to the powdered analogue (Figure 5).

The XRD spectrum measured for the rGO-powder, in a range of 2*θ* from 5° to 50° (Figure 4), shows a (002) diffraction peak at 2*θ* = 23.85°, indicating the distance between graphene layers, and a (10) diffraction peak at 2*θ* = 43.44°, indicating a short-range order in the stacked graphene layers. Bragg’s equation, Equation (2), was applied to the (002) reflection for evaluating the distance between graphene layers, denoted as *d* (Table 1).

Scherrer’s equation, Equation (3), using a constant equal to 0.9, was applied for the (002) reflection to evaluate the average height of the stacking layers, denoted as *H* (Table 1). Scherrer’s equation with a Warren constant of 1.84 [56,57] was applied to the 2D (10) reflection for estimating the average diameter of the stacking layers, denoted as *D* (Table 1). These calculations revealed that rGO consists of 4–5 graphene layers in a stacking nanostructure with an average diameter by height of about 4.11 × 1.79 nm, and a graphene layer distance of 0.37 nm. These values nicely correlate with the technical data sheet of rGO, reporting, on average, six layers of graphene (analytical methods: SEM and BET) distanced at 0.35 nm from each other.

The XRD patterns for the electrospun samples of all concentrations of rGO exhibit two strong diffraction peaks characteristic for the PEO crystalline structure at 2*θ* = 19.08° and 23.23° (Figure 4). The addition of a higher amount of rGO (≥5 wt%) led to the appearance of an additional broader halo peak (Figure 4). This halo peak centered around 2*θ*~23° corresponds to the (002) rGO maximum, and its exact position determines the distance between the graphene layers [58], which could serve as an indicator for a decrease in their ordering. Therefore, these broad peaks in the rGO-modified samples, with rGO content ≥ 5 wt%, were fitted by a Gaussian line shape to calculate the average distance between the graphene layers. The interlayer spacing, *d*, was calculated via Bragg’s equation, Equation (2), and the average height of stacking layers, *H*, was calculated via Scherrer’s equation, Equation (3), with a constant, dimensionless shape factor of 0.9. The calculated structural parameters of the electrospun PEO/rGO-based nanocomposites resulting from the XRD patterns are presented in Table 1. It is evident that the value of 2*θ* increased insignificantly (with the average distances between graphene layers decreasing accordingly) with the increase in the rGO concentration in the polymer system. The number of stacked layers decreased from 4–5 layers for the rGO-powder to 3–4 layers for the samples containing 5, 10 and 20 wt%. The absence of the halo peak at 2*θ*~23° for the samples with lower concentrations of rGO (≤1 wt%) indicates a total exfoliation of the rGO layers in the PEO matrix.

**Figure 6:**
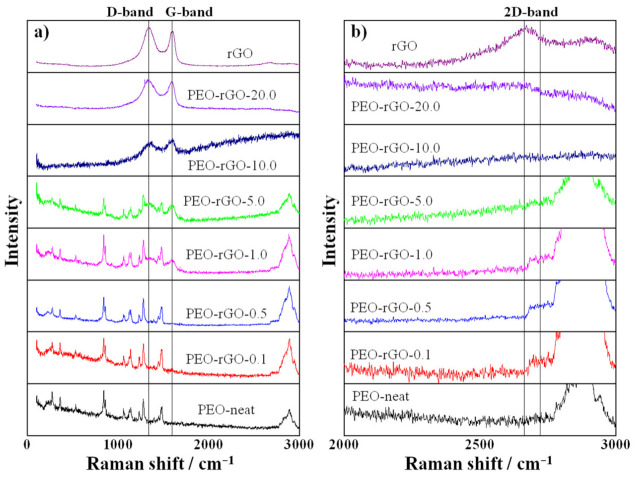
(**a**) Raman spectra of rGO, electrospun PEO-neat and electrospun PEO-based samples modified with rGO. (**b**) Zoomed spectral region to better visualize the appearance of the 2D band.

Raman spectroscopy offers a powerful capability to probe the structural characteristics and properties of graphene, graphene-based materials and their nanocomposites [59]. Here, the technique was utilized twofold: (i) to evaluate the presence of disorder in the *sp^2^* hybridization system, i.e., defects of the rGO sheets, and (ii) to determine the arrangement and the number of rGO layers. Figure 6 depicts the Raman spectra of rGO, electrospun PEO-neat and electrospun PEO-based samples modified with rGO.

The Raman spectrum of PEO is complex and rich in bands arising from the vibrations of the various molecular units within the polymer conformers. The bands at 1227 cm^−^^1^ and 1275 cm^−^^1^ are assigned to the CH_2_ wagging vibrations of trans- and gauche-conformers relative to the C–C bond, respectively, while the peaks at 1123 cm^−^^1^ and 1136 cm^−^^1^ arise from CH_2_ twisting vibrations of trans- and gauche-conformers relative to the C–O bond, respectively. The band at 1480 cm^−^^1^ is assigned to the CH_2_ bending vibrations, and the maximum at 844 cm^−^^1^ is attributed to the C–O stretching vibration and the CH_2_ rocking vibration [60]. Another intensive broad peak that appears at 2878 cm^−^^1^ is assigned to the symmetric C–H stretching vibration [61]. These PEO characteristic modes are present in the Raman spectra of the rGO-modified samples containing up to 5 wt% rGO, most probably because rGO at lower concentrations is majorly fully exfoliated in the PEO matrix. The bands from PEO are diminished in the Raman spectra of the samples containing the highest rGO concentrations, 10 and 20 wt%, because rGO at such high concentrations cannot be totally exfoliated, and the excess remains non-incorporated in the polymer matrix, thus resembling the spectral behavior of its initial, native compound. These considerations are agreeably explained by the manifestation of the D and G bands characteristic for the graphitic structure (G band at 1597 cm^−^^1^, emerging from the C–C vibrations of delocalized p-electrons with sp^2^ hybridization, and D band at 1335 cm^−^^1^, distinctive for the defects in the graphitic structures with sp^3^ hybridization [54,62]) that start to appear for the samples that contain 1 and 5 wt% rGO, alongside the bands characteristic for PEO. For higher rGO concentrations (10 and 20 wt%), as mentioned above, the spectral dominance features the rGO characteristic bands, D and G, completely suppressing the PEO bands (Figure 6, left panel).

The ratio between the intensity of the D and the G band (I_D_/I_G_) demonstrates the presence of defects in the graphene structure [63]. The Raman spectra of the pure rGO and all rGO-modified samples that exhibit D and G bands were curve fitted by a Gaussian function, and the peak intensity ratio I_D_/I_G_ was calculated. The value of I_D_/I_G_ for intact rGO was 1.21, presenting a low regime defect density. The increase in the rGO content (for PEO-rGO-20.0—1.05; for PEO-rGO-10.0—1.01; for PEO-rGO-5.0—1.05; and for PEO-rGO-1.0—1.06) returned a saturated value (around 1–1.05) indicating small defects in the graphitic structure for these electrospun samples. The important spectral feature should be noted for the samples with a lower rGO content (0.1; 0.5; and 1 wt%), where the 2D band around 2700 cm^−^^1^ emerged (Figure 6b). This 2D band originates from a two-phonon double resonance process and characterizes the arrangement and number of graphene layers [59,63], and its intensity is enhanced by reducing the number of graphene oxide layers [63]. The appearance of this band in the Raman spectra of the samples containing low concentrations of rGO (from 0.1 to 1 wt%) confirms the existence of an exfoliated structure for these samples, which is in good agreement with the XRD analysis results.

**Figure 7:**
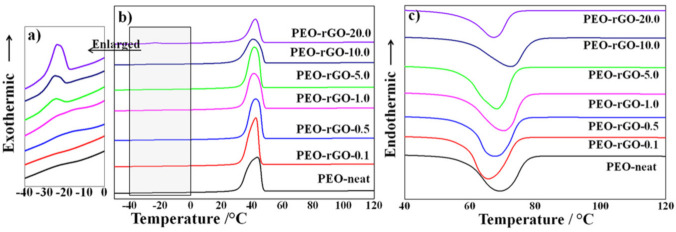
Nonisothermal DSC scans of PEO and rGO-modified samples at a constant scanning rate of 10 °C/min: (**a**) enlargement of the temperature sequence ~−25 °C, during cooling, where additional exothermic peaks appear, (**b**) cooling and (**c**) second heating.

DSC was employed to evaluate the effect of rGO on the phase transition behavior of PEO. Figure 7b,c depict, respectively, the first cooling and second heating thermograms of the PEO and rGO-modified electrospun samples.

The first cooling thermograms show that the peak crystallization temperature, T_c_, slightly downshifted (~3 °C) as the rGO concentration increased from 0.1 to 10 wt%, with PEO-rGO-10.0 showing the lowest crystallization temperature. Then, a slight upshift of 1.6 °C was observed for the sample with the highest rGO concentration (20 wt%). This non-monotonous trend of the crystallization temperature’s dependence on the rGO concentration can be explained by the competition between the nucleation and the confinement effect of the rGO nanolayers [61,64]. The addition of a well-dispersed small amount of rGO led to the formation of a rigid rGO network, which imposed a confinement effect on further crystal growth, similar to the case of CNT-modified polymer nanocomposites [64]. Subsequently, the crystallization temperature decreased. The upshift in the crystallization temperature for the sample with the highest rGO concentration (20 wt%) indicated that at this concentration, more polymer chains are in the vicinity of the rGO layers and nucleation therefore dominates the system. Interestingly, for the samples containing 5, 10 and 20 wt% rGO, additional exothermic peaks appeared at ~−25 °C in the cooling thermograms (Figure 7a).

The melting temperature (*T_m_*) exhibited a non-monotonous trend of dependence on the rGO concentration, and upshifts and downshifts in the *T_m_* with the rGO concentration could be observed (Table 2). On the contrary, the onset melting temperature (*T_m_^onset^*) was constant for almost all samples, meaning that the melting of PEO started at the same temperature for all samples regardless of the rGO content.

Obviously, there are two competing phenomena: (i) the increase in the thermal conductivity of the samples caused by the presence of rGO, which leads to a decrease in the melting temperature, and (ii) the formation of a rigid rGO network which imposes a strong confinement effect, and which leads to an increase in the melting temperature [65].

**Figure 8:**
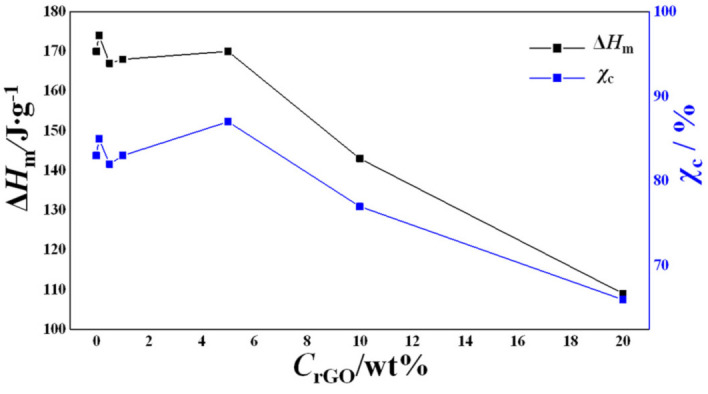
Dependence of enthalpy of fusion (Δ*H_m_*) and weight fractional crystallinities (*χ_c_*) on rGO content.

Figure 8 shows the dependence of the enthalpy of fusion (Δ*H_m_*) and the weight fractional crystallinities (*χ_c_*), calculated using Equation (5), on the rGO content.

A non-monotonous trend could be observed at lower concentrations of rGO, but there was a sharp decrease in Δ*H_m_* for the samples with 10 and 20 wt% rGO (Figure 8).

The change in the crystallinity with the increase in the rGO concentration may be attributed to the decrease in the mobility of the polymer chains in the electrospinning process due to the presence of rGO nanosheets. This effect is especially noticeable for the sample with the lowest diameter (as shown by SEM) containing 20 wt% rGO. Due to the large specific surface of nanofibers, the crystallization of the polymer in the process of electrospinning is hindered, and thus a polymer of lower crystallinity is obtained [66].

**Figure 9:**
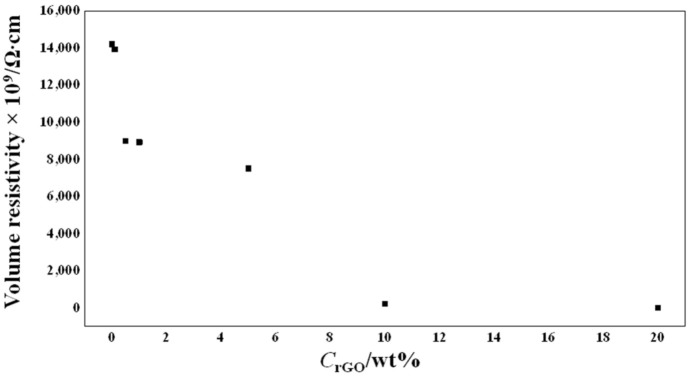
Electrical resistivity of the scaffolds as a function of the rGO content.

The electrical volume resistivity as a function of the weight content of rGO in the PEO matrix is presented in Figure 9. A significant reduction in the volume electrical resistivity of two orders of magnitude was obtained for the sample containing 10 wt% rGO, and a reduction of four orders of magnitude was obtained for the sample containing 20 wt% rGO. These noticeable reductions in resistivity, especially for the sample containing 20 wt% rGO, probably led to the significant reduction in the fiber diameter, determined by SEM morphological analysis.

**Figure 10:**
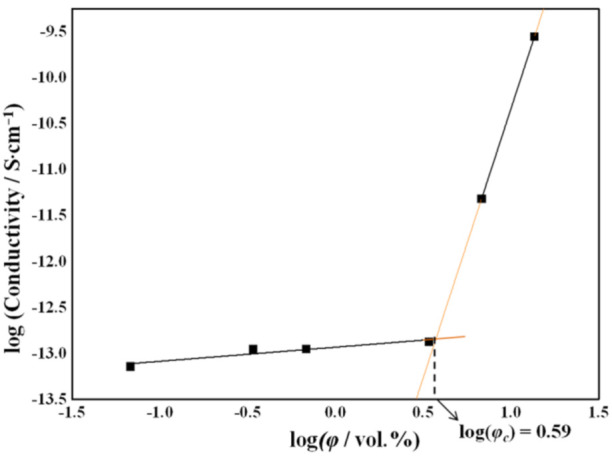
Double logarithmic plot of the conductivity as a function of the filler volume fraction.

A double logarithmic plot of the conductivity as a function of the filler volume fraction is shown in Figure 10. The experimental data can be linearly interpolated by two different straight lines, thus indicating the presence of two regimes below and above a critical filler concentration. The percolation threshold corresponds to the point where the two straight lines cross each other and was found to be 3.9 vol%, corresponding to a 5.7 wt% rGO concentration.

From the generalizations of the Bruggeman equation and a concept of shape-distributed particle composites, in 2D, the percolation threshold for identical overlapping ellipses with aspect ratio *η*, whose centers and orientations are random, can be fitted to the formula [65]

*ϕ_c_* = 3^4/(2+*η*+1/*η*)^. (1)

Taking the rGO particle size values obtained from the X-ray analysis, the calculated value for the critical filler volume fraction, *ϕ_c_*, is 3 vol%. This value is close to that estimated from the double logarithmic plot of the conductivity as a function of the filler volume fraction.

**Figure 11:**
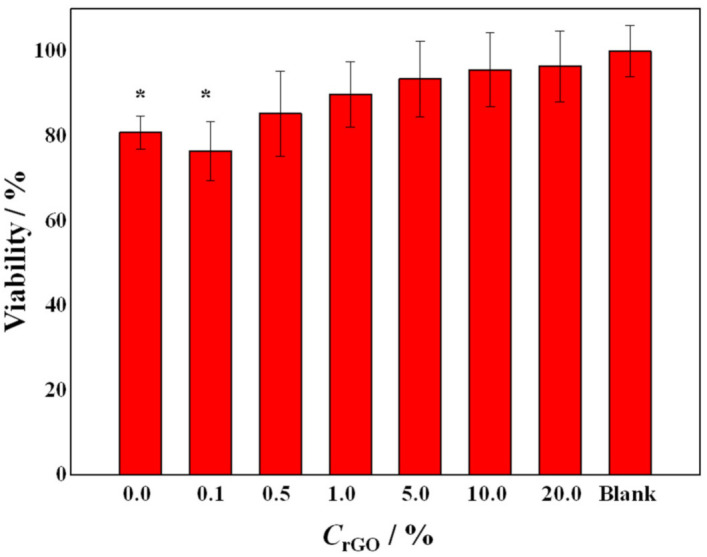
Viability of the cells treated with the liquid extracts from the electrospun samples with different rGO concentrations. The asterisk (*) represents a statistically significant difference (*p* < 0.01) relative to the untreated cells. The error bars present the standard deviation (*n* = 8).

The viability of epithelial colon cancer cells, SW-480 cell line, was assessed after a 48 h exposure to a liquid extract from the PEO/rGO samples (Figure 11).

The cells treated with liquid extracts from all samples did not demonstrate any signs of apoptosis or cell death (microscope observations); this, combined with the viability results from the MTT assay, points to the in vitro cell compatibility of the tested samples. There was an evident trend of increase in the cell viability with the increase in the rGO concentration in the PEO matrix, reaching 96% for the sample with the highest rGO concentration (20 wt%). The sample without rGO (POE-neat) and the sample that contained the lowest amount of rGO (0.1 wt%) demonstrated a statistically significant difference in viability (*p* < 0.01) relative to the untreated cells. The effects of PEO on similar cell culture lines showed that PEO markedly and dose-dependently inhibited their growth, and this cytostatic effect was associated with a blocking of the cell cycle in the G0/G1 phase [67]. However, the authors of the referenced study [67] did not find any evidence of programmed cell death (floating cells, DNA breakage, change in the phospholipid bilayer), which was the case in our experiments as well. The highest percentage of cell viability for the sample containing 20 wt% rGO could also be attributed to the re-agglomeration of the nanoparticles, as shown by SEM and Raman studies, and is in agreement with the size-dependent toxicity of carbon-based nanomaterials [68].

## 3. Materials and Methods

### 3.1. Materials

The polymer used in this study was SENTRY™ POLYOX™ WSR N80-NF GRADE, a water-soluble, non-ionic poly (ethylene oxide) polymer with a molecular weight of 200,000 Da, kindly supplied by Colorcon Limited, England. Reduced graphene oxide, partly reduced, 80% C in the form of powder, prepared by thermal reduction (flashing) of GO-powder, consisting of an average of 6 layers with a primary sheet thickness of 0.3 ÷ 0.4 nm, was kindly supplied by Abalonyx AS, Norway. Ethanol 96% Ph. Eur. was purchased from Alkaloid, Skopje.

### 3.2. Scaffold Production by Electrospinning

The typical horizontal setup of the electrospinning apparatus was used, which consisted of a high-voltage power supply (Genvolt HV power supply with (0 ÷ 30) kV power range, Bridgnorth, UK), a syringe pump (New Era Pump Systems, Farmingdale, NY, USA), a syringe with a needle (diameter 0.6 mm) and a rotating drum used as a collector. The high voltage was applied to the needle, and the drum was grounded. The scaffolds were collected as a fiber web on an aluminum foil substrate, which was attached onto the drum surface. The frequency of the drum rotation was kept constant at 300 min^−1^ for all samples.

For the obtainment of the neat PEO scaffold, a 10 wt% solution of PEO in a mixture of ethanol and deionized water (9:1 wt%:wt%) was prepared. The solution was homogenized by magnetic stirring overnight. For the obtainment of PEO/rGO scaffolds with different rGO concentrations (0.1, 0.5, 1, 5, 10 and 20 wt%, relative to the PEO), first, 0.001 g, 0.005 g, 0.01 g, 0.05 g, 0.1 g and 0.2 g of rGO were dispersed in 5 g ethanol using an ultrasonic bath with a frequency of 45 kHz for 10 min. Then, 1 g PEO, 4 g ethanol and 1 g deionized water were added into the dispersions, and magnetic stirring was applied overnight. Prior to electrospinning, all solutions containing rGO were ultrasonicated for 10 min in an ultrasonic bath with a frequency of 45 kHz. Immediately after sonication, the solutions were loaded in a plastic syringe connected to a 0.6 mm-diameter blunt-end needle, and the syringe was mounted on the digital syringe pump. Electrospinning was conducted at room temperature under standard atmospheric conditions. It was carried out using a voltage of 10 kV, a distance to the collector of 150 mm and a flow rate of 1.4 mL/h. The prepared samples were denoted as follows: PEO-neat, PEO-rGO-0.1, PEO-rGO-0.5, PEO-rGO-1.0, PEO-rGO-5.0, PEO-rGO-10.0 and PEO-rGO-20.0, according to their rGO concentrations.

### 3.3. Scanning Electron Microscopy (SEM)

Small cuts from each sample were mounted on a specimen stub with carbon tape. Then, the pieces were coated with an 8 nm layer of gold using a rotary pump sputter coater (Q150RS, Quorum Technologies, Laughton, UK). The morphology of the samples was studied using SEM (Merlin Gemini II, Zeiss, Oberkochen, Germany) at 2 kV, 100 pA. The working distance was optimized at 8–9 mm, and a secondary electron detector was utilized. The average fiber diameters were measured from 100 fibers based on 4 SEM images per sample using the ImageJ software (v. 1.51j8, Bethesda, MD, USA).

### 3.4. X-ray Diffraction

X-ray diffraction patterns were recorded on an Ultima IV diffractometer (Rigaku, Tokyo, Japan). The X-ray beam was Ni-filtered CuKα (λ = 0.154178 nm), and the radiation was generated by setting the tube voltage at 40 kV and the tube current to 40 mA. The scan rate of 5 °/min in a 2θ range from 5° to 50° was selected. The interlayer spacing was calculated via Bragg’s equation:

*λ* = 2*d*·sin*θ,* (2)

where *λ* is the X-ray wavelength of 0.154178 nm, d is the interlayer spacing and *θ* is the diffraction angle.

The average height of stacking layers, denoted as *H*, was evaluated using Scherrer’s equation:

*H* = (*K*λ)/(*β*cos*θ*), (3)

where *K* is a dimensionless shape factor with a value close to unity and has a typical value of about 0.9, *λ* is the X-ray wavelength, *β* is the peak full width at half maximum intensity (FWHM), after subtracting the instrumental line broadening (in radians), and *θ* is the Bragg angle. The diffraction (002) peak that evolved at 2*θ*~20° was used to determine the distance between graphene layers in each sample using Scherrer’s equation, Equation (3).

For estimating the average diameter of stacking layers, denoted as *D*, Scherrer’s equation with a Warren constant of 1.84 [56,57] was applied to the 2D (10) reflection. The peaks in each diffraction pattern were fitted by a Gaussian line shape from which the corresponding value for FWHM was obtained.

The degree of crystallinity was calculated from the area of crystalline peaks of diffraction *A_c_* and the area of amorphous peaks of diffraction *A_a_*, using Equation (4) [69]:

*χ_c_* = *A*_c/_(*A*_c_ + *A*_a_)*,* (4)

### 3.5. Raman Spectroscopy

The micro-Raman spectra of rGO embedded in the PEO electrospun samples were collected on a LabRam 300 spectrometer (Horiba Jobin Yvon, Piscataway, NJ, USA) using the 632.81 nm excitation line obtained from a He-Ne laser. A laser power of 0.11 mW was applied, and a ×50 objective for magnification was selected. The Raman peak shape of the D and G bands was fitted using a Gaussian function to obtain the *I_D_*/*I_G_* ratio in the rGO-modified samples. No spectral smoothing to improve the signal-to-noise ratio was applied.

### 3.6. Differential Scanning Calorimetry (DSC)

Thermal analysis of samples was conducted by using DSC (TA Instruments DSC Q20 V24, New Castle, PA, USA). The samples were loaded in an aluminum crucible under dry conditions; first, they were heated from room temperature to 150 °C, then cooled from 150 °C to −90 °C and then heated again from −90 °C to 150 °C. All the cycles were performed at a rate of 10 °C/min under a nitrogen atmosphere.

The melting temperature (*T_m_*), enthalpy of fusion (Δ*H_m_*) and weight fractional crystallinities (*χ*_c_, the degree of crystallinity of the PEO in the composites) were determined from the second heating curve. *χ_c_* was calculated using Equation (5):

*χ_c_* = (Δ*H*_m/_(Δ*H*^0^(1–*C*_rGO_))) × 100*,* (5)

where Δ*H*^0^ = 205 J/g is the enthalpy of fusion for 100% crystalline PEO [70,71], and *C*_rGO_ is the weight concentration of rGO present in the PEO matrix.

### 3.7. Electrical Resistivity Measurements

Structures used for measurement of electrical properties were formed with a dielectric film with a thickness d varying between *d* = (0.014÷0.004) mm for the PEO-rGO-5.0 sample and *d* = (0.038÷0.006) for the PEO-rGO-1.0 sample, slightly pressed between two flat plates of stainless steel: the bottom (substrate) and the upper electrode. The active cross-section of the formed resistors was *S* = 0.25 cm^2^. Based on a comparison of different section areas, it was concluded that the surface conduction is much lower than the bulk conduction, and hence the surface conductivity can be disregarded. Therefore, the resistance measured (*R*) can be expressed in terms of volume resistivity (*ρ*) as

*R* = *ρ*·*d/S,* (6)

The resistance of the structures (*R* = *U*/*I*) was determined based on the measurements of the current (*I*) obtained at a constant applied voltage (*U* = 1.0 V) using the HP 4140 B picoammeter/voltage source (Hewlett Packard, Palo Alto, CA, USA). In order to suppress the effect of displacement currents, there was a hold time of 5 s between the application of the voltage and measurement of the current.

### 3.8. Cell Culture

Epithelial colon cancer cells, SW-480 cell line (CLS GmbH, Kirkel, Germany) passage No 49–51, were used in the experiments. The cells were cultured in Ham F-12 medium (Sigma Aldrich, Baden-Württemberg, Germany) supplemented with 10% fetal calf serum (Gibco, North Lincolnshire, UK), streptomycin, amphotericin B and penicillin (Anti-Anti, Gibco, North Lincolnshire, UK) and maintained at 37 °C in an atmosphere of 10% CO_2_ and 95% humidity in a CO2 incubator (MRC, Holon, Israel). The cell culture passage was performed upon reaching 80% confluence.

### 3.9. In Vitro Cytotoxicity Experiments

The experiments were performed using liquid sample extracts. The liquid sample extracts were prepared by cutting a 3 × 3 cm piece from the electrospun patch of each sample, which was further dispersed in 5 mL complete cell culture medium in a sterile test tube. A test tube with 5 mL of medium was used as a blank sample. The samples were incubated for 24 h at 37 °C and afterwards centrifuged for 30 min at 2300× *g*. The extracted supernatants were further filtered through a 0.2 µm RC filter in aseptic conditions to remove any contaminants. The cells were seeded on a 96-well plate at a density of 5000 cells/well and left overnight to attach. Afterwards, the medium was aspirated and replaced with 0.2 mL/well of the prepared liquid sample extracts and blank sample. The cells were incubated for 48 h at 37 °C (10% CO_2_ and 95% humidity) and afterwards tested for viability using the standard MTT assay (Invitrogen, Carlsbad, CA, USA). In brief, 20 µL of MTT solution was transferred to each well, and the cells were incubated at 37 °C for an additional 4 h to allow the metabolic transition of MTT to the insoluble formazan. The medium was then removed, and dimethyl sulfoxide (DMSO) (0.2 mL/well) was added to dissolve the formazan crystals. The intensity of absorbance of each well was read at 560 nm using a multiplate reader, Victor X4 (Perkin Elmer, Waltham, MA, USA). The cell viability (%) was calculated as the ratio of each sample to the control (untreated) group.

## 4. Conclusions

Within this study, electrospun PEO-based scaffolds with different concentrations of rGO (from 0.1 to 20 wt%) were formulated. PEO was selected due to its ability to suppress the cytotoxicity of carbon-based nanofillers and its high electro-spinnability. The effect of the concentration of rGO in PEO fibers was evaluated in terms of morphology and structural changes, as well as electrical properties and cytotoxicity. Characterization was performed using Raman spectroscopy, XRD, SEM, DSC and electrical measurements. In vitro cytotoxicity experiments were performed on epithelial colon cancer cells. The results show a good dispersion of rGO up to a concentration of 1 wt%, a drastic reduction in the fiber diameter for the highest concentration of 20 wt% and, at the same time, a drastic decrease in the electrical resistivity. Furthermore, an evident trend of increasing cell viability as the rGO concentration increased was noted, reaching 96% for the highest rGO-loaded sample (20 wt%). These promising results can serve as a pathway for the judicious selection of the optimal rGO amount incorporated in the PEO matrix when constructing electroactive scaffolds.

## Figures and Tables

**Table 1:** Structural parameters of the rGO-powder and the electrospun PEO/rGO-based nanocomposites resulting from the XRD patterns. Notation: *H*—average height of the stacking nanolayers; *n*—average number of graphene layers in graphene stacking nanolayers; *d*—average distance between graphene layers; *D*—average diameter of rGO stacking nanolayers.

Sample	Peak (002)				
	2*θ*/°	FWHM/°	*H*/nm	*d*/nm	*n*
PEO-rGO-5.0	21.58	5.96	1.43	0.41	3–4
PEO-rGO-10.0	21.61	6.13	1.31	0.41	3–4
PEO-rGO-20.0	22.07	5.52	1.47	0.40	3–4
rGO	24.32	4.53	1.79	0.36	4–5
**Sample**	**Peak (10)**				
	**2*θ*/°**	**FWHM/°**		***D*/nm**	
rGO	43.44	4.24		4.11	

**Table 2:** Crystallization and melting temperatures for PEO-neat and rGO-modified samples.

Sample	First Cooling		Second Heating	
	*T_c_*^′^/°C	*T_c_*^″^/°C	*T*_m_^onset^/°C	*T_m_*/°C
PEO-neat	46	/	58	69
PEO-rGO-0.1	42	/	58	66
PEO-rGO-0.5	42	/	60	68
PEO-rGO-1.0	41	/	58	70
PEO-rGO-5.0	42	−25	58	68
PEO-rGO-10.0	41	−25	58	72
PEO-rGO-20.0	42	−24	58	67

## Data Availability

All data in this study can be requested from the corresponding author (aivanoska@manu.edu.mk).
